# Why I cannot return home yet

**DOI:** 10.7554/eLife.92566

**Published:** 2023-09-14

**Authors:** Mariano A Molina

**Affiliations:** 1 Amsterdam University Medical Center, Vrije Universiteit Amsterdam Amsterdam Netherlands; 2 Imaging and Biomarkers program, Cancer Center Amsterdam Amsterdam Netherlands

**Keywords:** sparks of change, inclusion, self-acceptance, research culture, researcher mobility, identity

## Abstract

Studying abroad helped a Panamanian student to accept who he is, but it meant him letting go of his dream.

Packing my luggage for my first trip back home in more than two years, my emotions were mixed. I was excited to finally see my friends and family in Panama after studying abroad in the Netherlands for so long. I was also deeply upset that I would be returning alone. However, as much as I wanted my partner to finally meet my family, the stigma and discrimination against LGBTQIA+ people in Panama made it unsafe for us to travel there together.

This was especially difficult for me to accept because I always felt a strong sense of patriotism. I take my official Panamanian football shirt with me on almost every trip and have only happy memories of the celebrations held each November for “Mes de la patria” or “patriotic month”. Still, as much as I loved my home country, growing up in Panama had not helped me to love and accept myself for who I am.

Throughout my life, I had witnessed people – including some of those close to me – harassing or looking down on others simply for being gay, lesbian or trans. Hurtful slurs were often used without a second thought. To this day, education about gender identity or sexuality remains almost non-existent in Panama and prejudices based on religion or personal upbringing are commonplace. Most LGBTQIA+ Panamanians as a result grow up not truly knowing about their own identities; this is also what happened with me.

Looking back, I knew that I did not feel like everyone else. Yet in moments when I had considered who I might be and certain labels came to mind, I did not feel like any of them either. As such, because at that time I was not sure about it myself, I left Panama aged 24 without ever having told anyone else that I was queer.

Before I left, I remember speaking with a colleague at my farewell party who had also studied abroad in Europe, specifically in Germany. She told me that I could essentially become anyone I wanted because I would be in a new place and make new friends. That conversation stuck with me. On some level, I see it was the Netherlands’ reputation for being an inclusive and tolerant society that drew me to apply there for my studies. My patriotism, nevertheless, meant I still dreamed of eventually returning to find a job at one of the scientific institutes in Panama City that I so admired, to do science both in and for Panama.

During the introduction week of my master’s degree in microbiology at Radboud University in the Dutch city of Nijmegen, I was struck by how diverse a group the students there were. Later, when I saw LGBTQIA+ people doing simple things like holding hands, taking selfies together, or going out on dates, it just felt right.

Within my first few months, I met another student on a course about medical microbiology, who was very smart and kind. He and I got talking and, two months later, we were officially dating. We have been together ever since. As we grew closer, I shared my thoughts, fears and aspirations with him as someone who both valued and respected me; this played a crucial role in me understanding who I was and in boosting my self-confidence. Later, with much anticipation and anxiety, I told my family and friends about my identity too. I feel fortunate that most of my loved ones responded with understanding and support. I also spoke with a therapist who helped me through the process by teaching me to navigate my emotions, confront my insecurities, and develop some coping strategies.

Earlier this year, my partner and I got married at a small ceremony in Utrecht after which we had dinner with our friends. Together we celebrated my first Pride in Utrecht in June and joined the Pride Boat Parade in Amsterdam in August as well. As I danced on that boat, with over 50 other people and everyone around the canals watching and dancing too, it was one of the first times in my life that I expressed myself for who I am without also feeling fear.

While my partner could not come with me on that trip back home in March 2022, we are planning a trip to Costa Rica where it will be safer for us to travel together. Some family members will also come to the Netherlands for my PhD defense in a few months’ time, which will be a better option for them to meet him too.

As my acceptance of my own identity as a queer Latino grew, my priorities shifted in parallel. I had accepted that it would not be safe for neither me nor my partner to live in Panama, so I also let go of my dream to return home to work there immediately after my studies. Instead, I am now a postdoc at the Amsterdam University Medical Center and my partner is a doctoral candidate at the Radboud University Medical Center. I do, however, want to get affiliated with a Panamanian institution in some way and do research remotely that would still help advance science in my home country. I also feel passionate that more should be done for displaced LGBTQIA+ scientists who, like me, have decided to stay abroad due to the risk of violence and discrimination in their native countries. I am sure that I am not alone, and I am motivated to help the community that I now truly feel a part of.

Finally, while it may take several more years to see a real change, the activities of some advocacy organizations back home (like Fundación Iguales and Ciencia en Panamá) give me hope. I remain optimistic that one day everyone in Panama will be lawfully protected and feel safe, and that my dream of living and working in science in my home country will still come true.

## Share your experiences

This article is a Sparks of Change column, where people around the world share moments that illustrate how research culture is or should be changing. Have an interesting story to tell? See what we’re looking for and the best ways to get in touch here.

